# Early-Life Development of the Bifidobacterial Community in the Infant Gut

**DOI:** 10.3390/ijms22073382

**Published:** 2021-03-25

**Authors:** Silvia Saturio, Alicja M. Nogacka, Marta Suárez, Nuria Fernández, Laura Mantecón, Leonardo Mancabelli, Christian Milani, Marco Ventura, Clara G. de los Reyes-Gavilán, Gonzalo Solís, Silvia Arboleya, Miguel Gueimonde

**Affiliations:** 1 Department of Microbiology and Biochemistry of Dairy Products, IPLA-CSIC, 33300 Villaviciosa, Spain; Silvia.Saturio@ipla.csic.es (S.S.); alicja.nogacka@ipla.csic.es (A.M.N.); greyes_gavilan@ipla.csic.es (C.G.d.l.R.-G.); 2 Diet, Microbiota and Health Group, Instituto de Investigación Sanitaria del Principado de Asturias (ISPA), 33011 Oviedo, Spain; nuriajmhd@gmail.com; 3 Pediatrics Service, Hospital Universitario Central de Asturias, SESPA, 33011 Oviedo, Spain; msr1070@hotmail.com (M.S.); laura_mantecon@hotmail.com (L.M.); gsolis@telefonica.net (G.S.); 4 Pediatrics Research Group, Instituto de Investigación Sanitaria del Principado de Asturias (ISPA), 33011 Oviedo, Spain; 5 Pediatrics Service, Hospital de Cabueñes, SESPA, 33203 Gijón, Spain; 6 Laboratory of Probiogenomics, Department of Chemistry, Life Sciences and Environmental Sustainability, University of Parma, 43121 Parma, Italy; leonardo.mancabelli@unipr.it (L.M.); christian.milani@unipr.it (C.M.); marco.ventura@unipr.it (M.V.)

**Keywords:** *Bifidobacterium*, infant, microbiota, gut, preterm, delivery mode, feeding

## Abstract

The establishment of the gut microbiota poses implications for short and long-term health. *Bifidobacterium* is an important taxon in early life, being one of the most abundant genera in the infant intestinal microbiota and carrying out key functions for maintaining host-homeostasis. Recent metagenomic studies have shown that different factors, such as gestational age, delivery mode, or feeding habits, affect the gut microbiota establishment at high phylogenetic levels. However, their impact on the specific bifidobacterial populations is not yet well understood. Here we studied the impact of these factors on the different *Bifidobacterium* species and subspecies at both the quantitative and qualitative levels. Fecal samples were taken from 85 neonates at 2, 10, 30, 90 days of life, and the relative proportions of the different bifidobacterial populations were assessed by 16S rRNA–23S rRNA internal transcribed spacer (ITS) region sequencing. Absolute levels of the main species were determined by q-PCR. Our results showed that the bifidobacterial population establishment is affected by gestational age, delivery mode, and infant feeding, as it is evidenced by qualitative and quantitative changes. These data underline the need for understanding the impact of perinatal factors on the gut microbiota also at low taxonomic levels, especially in the case of relevant microbial populations such as *Bifidobacterium*. The data obtained provide indications for the selection of the species best suited for the development of bifidobacteria-based products for different groups of neonates and will help to develop rational strategies for favoring a healthy early microbiota development when this process is challenged.

## 1. Introduction

The gastrointestinal tract is home to the intestinal microbiota that constitutes a very rich and complex microbial ecosystem. In human adults, this microbiota is dominated by the phyla Firmicutes and Bacteroidetes. However, during early life, the microbiota is mainly constituted by Actinobacteria and Proteobacteria, becoming more diverse later on with the rise of Firmicutes and Bacteroidetes. The establishment of this microbiota starts at birth when a massive exposure to microbes begins, making possible the extensive colonization of the newborn [1]. This process of early microbiota establishment plays a critical role in the promotion of the immune and physiological homeostasis, strengthening of intestinal barrier function, and, therefore, the health of the infant. There is accumulating evidence showing that early life microbiota establishment is a key determinant for later health [2,3,4,5,6,7,8,9]. In humans, early-life microbiota alterations have been linked to higher risks of disease in later life [10,11,12,13]. These underline the importance of understanding this process and suggest that any alteration may have implications for immediate and long-term health.

The colonization of the neonatal gut starts with the establishment of facultative anaerobic and aerotolerant microbial populations that, by reducing the environment together with the antioxidant systems of the newborn, allow the progressive establishment of strict anaerobes mainly from the genera *Bifidobacterium* and *Bacteroides* [1]. After these initial colonization steps, in the case of healthy full-term breast-fed infants, the microbiota will soon become dominated by Actinobacteria, mainly from the genus *Bifidobacterium*, with relatively high levels of Proteobacteria as well [1]. Bifidobacteria harbor different saccharolytic capabilities, allowing these microorganisms to metabolize carbohydrates, either from the diet or those of host origin [14,15,16]. Among the latter, the ability of some species of bifidobacteria to metabolize human milk oligosaccharides is an important characteristic that contributes to their dominant role on the breast-fed infant microbiota [17,18]. Moreover, bifidobacteria are considered beneficial microorganisms, and reduced levels have been often associated with disease [19,20]. This seems to be especially true in the case of infants where reduced levels of *Bifidobacterium* have been related to different conditions, such as allergic diseases or obesity [10,11,12]. Moreover, not just the alteration on the total *Bifidobacterium* numbers but also on the profile of species present have been reported to be of relevance [20,21]. Interestingly, some species such as *Bifidobacterium 14* or *Bifidobacterium catenulatum* are more abundant in adults. Other species, including *Bifidobacterium bifidum* or *Bifidobacterium breve,* are more numerous in infants, with *Bifidobacterium longum* being the species most widely present across life [22].

During the last years, we have learned that the intestinal colonization process depends on several factors, both of genetic and environmental origin. The impact of many of these factors on the global microbiota composition has been studied. Today we know that different early-life factors, including gestational age at birth, mode of delivery, feeding habits, etc., affect the process of establishment of the intestinal microbiota in the newborn [1,23]. Some of these factors, such as cesarean section (CS) delivery, are associated with increased disease risk [24]. Most of the available data relies on the use of 16S rRNA gene-based sequencing, which provides an overview of the global microbiota composition, and has demonstrated delayed colonization by bifidobacteria in preterm babies or after CS, among others [23,25,26]. However, this technique does not provide detailed information at low taxonomic levels, such as species and subspecies. Therefore, our understanding of the impact of early life factors upon the establishment of relevant intestinal populations, such as the bifidobacterial community, remains limited. Most of the quantitative information available regarding the bifidobacterial species composition in infancy comes from PCR and hybridization-based techniques [27,28,29,30], with very limited data available regarding the very early stages of life. To overcome these limitations, other methodological approaches should be used. In this regard, the Internally Transcribed Spacer (ITS) sequence, the region between the 16S rRNA and the 23S rRNA genes within the rRNA locus, has demonstrated to be an applicable marker for *Bifidobacterium* species, and an ITS-based protocol (ITS-*Bifidobacterium* profiling) has been developed to this end [31]. This ITS-based protocol has been used to track the vertical transmission of bifidobacteria [32,33] or the effect of donor versus own-mother milk on the bifidobacterial community in premature babies [34]. In this context, we hypothesized that, as occurs with the global microbiota composition, the pattern of establishment of the bifidobacterial populations in the neonate is affected by perinatal factors such as prematurity, delivery mode, or infant feeding mode.

In this study, we aimed at determining the pattern of establishment of the intestinal bifidobacterial population in early life. To this end, we took advantage of the above-mentioned ITS-*Bifidobacterium* profiling and of qPCR for specific bifidobacterial species for assessing the bifidobacterial microbiota composition in newborns during the first three months of life. We evaluated the impact of prematurity, CS, and feeding regime upon the *Bifidobacterium* population development.

## 2. Results

### 2.1. Bifidobacterium Community Development in Newborns

**Figure 1:**
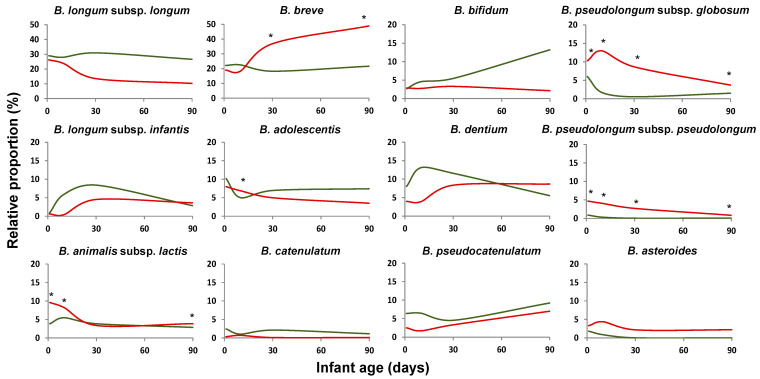
Impact of prematurity. Average relative proportions, with regard to total bifidobacteria (100%), of the dominant *Bifidobacterium* species/subspecies (based on the sequencing of the internal intranscribed spacer (ITS)-region between the 16S and 23S rRNA genes) during the first three months of life in full-term (green line) and preterm (red line) newborns. * Indicates statistically significant differences (*p* < 0.05) at the corresponding sampling times (two, 10, 30, or 90 days of age).

Several *Bifidobacterium* species were already detected from the second day of life, and the levels of the different species changed over time. *B. longum* and *B. breve* were the species showing higher occurrence on the second day of life (97.5% and 96.5 %, respectively) being detected in 99% of the babies at three months of age (Supplementary Table S1). These two species were also the ones found to be present at higher relative proportions and showing higher absolute levels (Figure 1 and Supplementary Figure S1). The detection rates for *B. bifidum* ranged from 79% of babies at two days of age and 89% at three months. Other than these general observations, clear differences in the bifidobacterial community composition and levels were observed among different infant groups, as is depicted below.

### 2.2. Bifidobacterium Species Composition Is Affected by Prematurity

Preterm delivery was found to have an effect, at both qualitative and quantitative levels, on the development of the bifidobacterial population in the newborn during the first months of life. Preterm infants presented an increased number of coexisting species, with an average of 19 ± 9 (mean ± sd) different species/subspecies found in contrast to the 12 ± 6 species/subspecies co-occurring, on average, in term babies. These figures remained stable during the three months of the study, with the differences reaching statistical significance (*p* < 0.05) at the four-time points analyzed (two, 10, 30, and 90 days of age) (data not shown). In addition, the relative proportions of the dominant bifidobacterial species were clearly different between both groups of infants. Full-term babies harbored higher early life proportions of *B. longum* and *B. bifidum* and lower of *B. breve*, *Bifidobacterium pseudolongum*, *Bifidobacterium asteroides*, and *Bifidobacterium animalis* subsp. *lactis*, among others (Figure 1, Supplementary Table S2). These observations were further confirmed when the absolute levels of *B. longum*, *B. bifidum*, *B. catenulatum*, *Bifidobacterium dentium*, *B. adolescentis*, and *B. breve*, were determined by qPCR (Supplementary Figure S1). Counts of total bifidobacteria also showed that premature babies harbored lower total *Bifidobacterium* levels.

**Figure 2:**
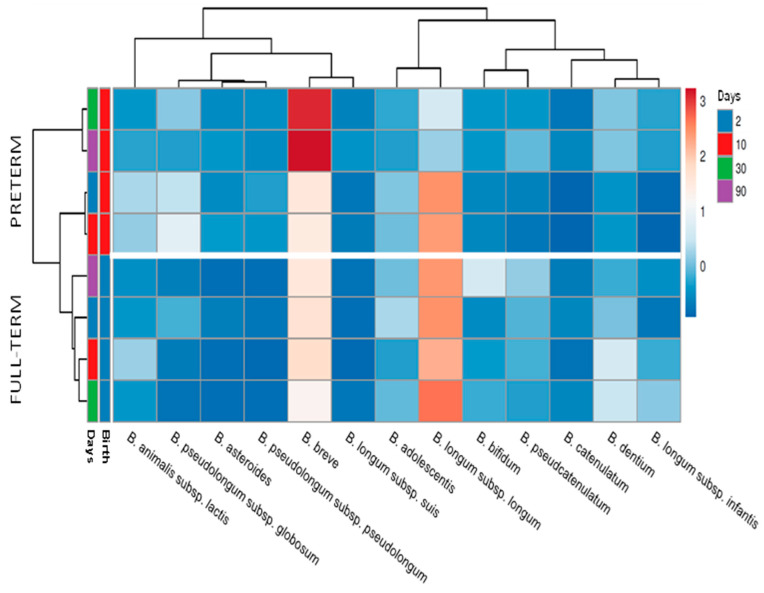
Heatmap showing the relative proportions of the different *Bifidobacterium* species/subspecies at two, 10, 30, and 90 days of age in full-term and preterm babies. The scale goes from blue (low relative frequency) to red (high relative frequency). Species/subspecies representing less than 1% were excluded from the analysis.

Interestingly, whereas in full-term babies, *B. longum* subsp. *longum* was the dominant bifidobacteria during the whole duration of the study, in preterm infants, this microorganism dominated initially (two and 10 days of age), but the dominance switched towards *B. breve* at later time points (30 and 90 days of age) (Figure 2), as was also confirmed by qPCR (Supplementary Figure S1).

The large differences existing between these two groups of infants precluded us from combining them, and, therefore, they were considered as two independent groups for any further analyses.

### 2.3. Mode of Delivery Affects the Bifidobacterial Population Establishment in Early-Life

The diversity of bifidobacteria, measured as the number of species simultaneously co-occurring in the infant, was not affected by the type of delivery either in full-term or in preterm newborns at any of the analyzed time points (*p* > 0.05). The mean values ranged from 11 to 13, depending on the sampling time, in vaginally delivered full-term babies and from 12 to 14 in CS delivered ones. In preterm infants, the mean values of species per sample moved between 21–22 during the first three months of life in vaginally-delivered babies and from 16 to 19 in CS premature infants.

**Figure 3:**
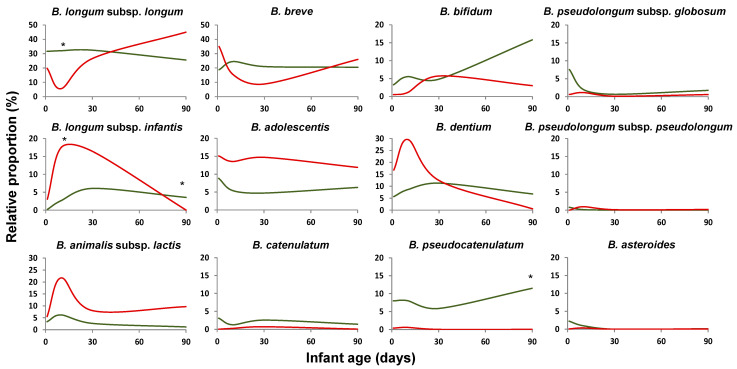
Impact of delivery mode. Average relative proportions, with regard to total bifidobacteria (100%), of the dominant *Bifidobacterium* species/subspecies (based on ITS-sequencing) during the first three months of life in full-term babies delivered vaginally (green line) or by cesarean section (CS) (red line). * Indicates statistically significant differences (*p* < 0.05) at the corresponding sampling time (two, 10, 30, or 90 days of age).

In full-term newborns, the delivery mode was found to affect the establishment of the bifidobacterial community. Among the dominant *Bifidobacterium* species/subspecies, CS-delivery led to an initial reduction in the relative proportions of *B. longum* subsp. *longum*, *B. bifidum*, or *Bifidobacterium pseudocatenulatum*, but increased those of *B. adolescentis*, *B. animalis* subsp. *lactis*, *B. dentium*, or *B. longum* subsp. *infantis* (Figure 3), without detecting statistically significant differences for any of the minority species (Supplementary Table S3). After three months, some of these differences, such as those observed for *B. longum*, seemed to be reduced. The qPCR analyses confirmed some of the previous findings, with higher levels of *B. longum* (*p* < 0.05) in CS-babies at three months of age (Supplementary Figure S2).

In preterm newborns, the delivery mode did not show any major effect on the bifidobacterial community. The only statistically significant (*p* < 0.05) difference observed among the dominant species was a reduced proportion of *B. bifidum* at an early age (two days) in CS babies. Some differences were also observed for some of the minority species (Supplementary Table S4).

### 2.4. Impact of the Feeding Mode on the Bifidobacterial Population in Early-Life

In order to ascertain the effect of feeding type on the establishment of the bifidobacterial population and to avoid the impact of potentially confounding factors such as delivery mode, we decided to focus this analysis on full-term vaginally delivered infants (34 out of the 43 full-term infants included in the study). Preterm infants were not included since the categorization of these infants according to feeding mode resulted in uneven groups and difficulties for a straightforward classification, since most babies received nutritional enrichment, mixed feeding, etc., during the course of the study, which could hamper the strength of the conclusions.

**Figure 4:**
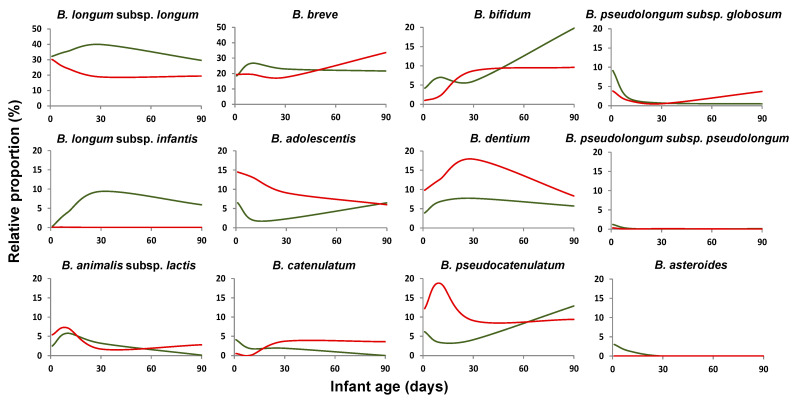
Average relative proportions, with regard to total bifidobacteria (100%), of the dominant *Bifidobacterium* species/subspecies during the first three months of life in exclusively breast-fed full-term vaginally delivered (green line) and formula/mixed-fed full-term vaginally delivered babies (red line).

As with regard to the results obtained for the full-term vaginally delivered babies, no differences in the number of detected *Bifidobacterium* species were observed between exclusively breast-fed and formula/mix-fed groups (ranging between 12 and 14, depending on the sampling time). In this group, exclusive breast-feeding was found to promote higher relative proportions of *B. longum* subsp. *longum*, and *B. longum* subsp. *infantis* slightly (0.05 < *p* < 0.1) during the first three months of life, and *B. bifidum* at the end of this period. On the contrary, formula/mix feeding promoted *B. adolescentis*, *B. pseudocatenulatum*, and *B. dentium* during the first days of life and *B. breve, B. catenulatum* and *B. animalis* subsp. *lactis* after three months (Figure 4, Supplementary Figure S3). These data were confirmed when the absolute levels of these bifidobacterial species were determined by qPCR (Supplementary Figure S4). The levels of *B. adolescentis* were higher (*p* < 0.05) during the first month of life, and those of *B. catenulatum* and *B. animalis* were higher at three months of age (*p* < 0.05) in formula/mix fed babies compared to exclusively breast-fed ones.

## 3. Discussion

During the last decade, we have started to understand the critical role of the early microbiota for the later health of individuals [5,35]. However, our knowledge about the early microbiota composition at low taxonomic levels is still limited. This is especially relevant for some microorganisms, such as the *Bifidobacterium* genus, since these microorganisms are dominant during early life and have often been linked to a healthy status [16].

Several studies have reported the effect of gestational age and preterm delivery, on the development of the microbiota, at a general level by using 16S rRNA gene-based sequencing [36,37,38,39,40]. Although some studies have reported low levels or no bifidobacteria in the infants’ gut [41,42], this has been linked to potential methodological biases or geographical differences [43], whilst most of the studies have found *Bifidobacterium* among the dominant microbial genera in infants. However, limited information is still available at the bifidobacterial community level. In general, the dominant bifidobacterial species identified in the present study are in good agreement with those previously indicated as dominant within this genus [31,44]. Here, we observed a clearly different pattern, at both qualitative and quantitative levels, for the development of the *Bifidobacterium* population in preterm when compared with full-term newborns. The higher number of bifidobacterial species found in premature with respect to full-term babies along the studied period results intriguing since previous studies reported a reduced global microbiota diversity in preterm babies or the lack of diversity differences between terms and preterms [45,46]. This contrasts with the situation in adults where increased microbiota diversity is generally considered beneficial and associated with reduced disease risk [47]. However, our findings are in good agreement with other studies in which high bifidobacteria diversity has been reported in situations where the scenario for the gut microbiota development is not ideal [34].

Our data suggest that the differences in the dominant bifidobacterial species/subspecies between full-term and preterm babies do not lie in the very early days but rather become larger at slightly later stages. At early time points, *B. longum* was the dominant species, in both preterm and full-term groups and remains so in full-term babies. However, in preterm babies between 10 and 30 days of life, there was a switch to *B. breve* becoming the dominant species. Although the reason for this is not understood, it suggests that differences in the management of both groups of infants, home versus hospital environment, medicalization, etc., may partly account for the observed differences. Indeed, the levels of the genus *Bifidobacterium* have been one of the microbiota characteristics more deeply affected by prematurity in different previous studies [25,38,40]. Our results extend these observations to the levels of specific *Bifidobacterium* species and to the global bifidobacterial community. In accordance with our results, other authors have demonstrated higher absolute levels of *B. longum* in full-term than in preterm babies during the first months of life, as is the case for one-month-old Brazilian infants [48].

Delivery mode is another of the perinatal factors known to affect the general profile of the microbiota [49,50], and in this regard, our data indicate that the bifidobacterial community is not an exception, and it is also affected by this factor. This holds true for full-term infants where the differences observed between vaginally and CS-delivered babies were of relevance; this is in good agreement with previous studies, based on other techniques, that also observed an impact of delivery mode on intestinal bifidobacteria. In agreement with our observations, Yang et al. [44], using a sequencing strategy based on the *gro*El gene, found that six-week-old CS-delivered full-term infants had higher proportions of *B. longum* subsp. *infantis* and *B. animalis* subsp. *lactis* than their vaginally-delivered counterparts. Interestingly, this does not seem to be the case for *B. longum* subsp. *longum*, whose relative proportions, according to our results, were higher in vaginally delivered babies during the first month of life and lowered later on. In this regard, qPCR-based studies have often reported higher levels of *B. longum* in vaginally delivered full-term babies [30,48]. It has to be taken into account that qPCR renders absolute quantitative values and often quantifies the different subspecies of *B. longum* together, which may explain the differences observed when comparing with sequencing data. The results obtained by us regarding other species such as *B. bifidum* or *B. pseudocatenulatum* also confirm those previously obtained by qPCR in other studies [30]. Moreover, Backhed and coworkers [50], using shotgun metagenomic analyses, found that CS-delivered babies, in agreement with our results, harbored lower proportions of *B. longum* or *B. catenulatum* and higher of *B. animalis* subsp. *lactis*. However, these authors reported reduced levels of *B. adolescentis* in these CS-delivered babies, whereas we found the opposite effect.

The differences due to delivery mode observed in full-term babies do not seem to be present in preterm babies since only minor differences related to delivery mode were found in our cohort by ITS-sequencing and by qPCR. Similarly, Grzeskowiak and coworkers [48], using qPCR, only observed an increase in the levels of *B. animalis* subsp. *lactis* in CS-delivered preterm babies without differences among the species analyzed. This is very likely due to the several factors affecting the microbiota development in preterm babies, from prematurity itself to medicalization, management in a hospital environment, etc. In this context, in preterm infants, the contribution of the delivery mode to the establishment of the bifidobacterial microbiota seems to be lower in comparison with those other factors.

Moreover, the mode of delivery did not affect the number of species of bifidobacteria present, not in full-term neither in preterm infants. This is in agreement with previous studies evidencing the lack of effect of delivery mode upon the total microbiota diversity in the sort and the long term [44,51].

As with regard to the feeding mode, our data suggest a moderate effect of this factor on the bifidobacterial microbiota of vaginally delivered full-term babies. This was in contrast with the high impact of feeding habits reported by some authors on the global microbiota composition or on the *Bifidobacterium* species [27,50,52], whereas some other studies have also reported limited effects of the feeding habit on the microbiota. These differences among studies may be partly due to the difficulties for the classification of the feeding habit, since in some cases, like in this study, exclusively breast-fed babies were compared with non-exclusively breast-fed babies, whereas other studies consider infants breast-fed, although not exclusively, in comparison with those receiving exclusively formula. In accordance with a previous report [30], we observed reduced levels of *B. adolescentis* or *B. dentium* in exclusively breast-fed newborns. On the contrary, we found increased levels of *B. longum* in these infants, although this has not been observed in other studies [30,44]. However, it is important to take into account that in the present work, the number of infants not being exclusively breast-fed was low (*n* = 10), with most of them being in mixed feeding, which does not allow accounting for the actual amount of formula/breast-milk received.

To sum up, this study underlines the process of development of the bifidobacterial microbiota in the newborn during the first months of life and the factors driving it.

## 4. Materials and Methods

### 4.1. Volunteers and Sampling

The study was approved by the Regional Ethical Committee of Asturias Public Health Service (SESPA), and informed written consent was obtained from each infant’s parents. The study included 85 healthy neonates (Table 1), not receiving pro- or pre-biotics during the sampling time, born at the Central University Hospital of Asturias (Northern Spain).

Fecal samples were collected at 2 (between 24 and 48 h of life), 10, 30, and 90 days of age. The sample was taken in a sterile container and immediately frozen at −20 °C until delivery to the laboratory for further analyses.

### 4.2. Faecal DNA Extraction

For DNA extraction, the fecal samples were allowed to thaw at room temperature (20–22 °C). Then 1 g of sample was weighed, diluted 1:10 in sterile PBS solution, homogenized in a LabBlender 400 stomacher (Seward Medical, London, UK) at full speed for 3 min, centrifuged, and the bacterial pellet obtained was used for DNA extraction as previously described [25]. Extracted DNA was kept frozen at −70 °C until analysis.

### 4.3. ITS Sequence-Based Bifidobacterial Microbiota Analysis

Fecal bifidobacterial population profiling was carried out by ITS-*Bifidobacterium* sequencing as previously described [31]. In brief, the bifidobacterial ITS region was amplified by using the primer pairs Probio_bif_uni and Probio_bif_rev; the amplicons were subjected to next-generation sequencing and sequences filtered and annotated using an improved bifidobacterial ITS database and a custom bioinformatics script, as described by Milani and collaborators [31].

### 4.4. Analyses of Faecal Bifidobacterial Levels by qPCR

The levels of total *Bifidobacterium* as well as those of the species *B. bifidum*, *B. breve*, *B. catenulatum*, *B. dentium*, *B. longum*, *B. angulatum*, *B. animalis* subsp. *lactis*, and *B. adolescentis* were determined by qPCR using the methods described elsewhere [25,34].

### 4.5. Statistical Analyses

Results were analyzed using the SPSS software version 26 (SPSS Inc., Chicago, IL, USA). The normality of the qPCR data, at each sampling point, was checked using the Kolmogorov–Smirnov test. Some of the bacterial groups showed non-normal distribution, and therefore, differences in bacterial levels and abundances between groups of infants were analyzed using non-parametric tests (Mann–Whitney U-test). Heatmaps were obtained by using the relative abundance data of the bifidobacterial species, filtered by a minimum presence ≥ 1, by using ClustVis [53].

## Figures and Tables

**Table 1:** Basal characteristics of the infant groups included in this study.

	Full-Term Babies (*n* = 43)	Preterm Babies (*n* = 42)
Weeks of gestation (mean ± sd)	39 ± 2	31 ± 2
Gender (*n* females)	22	18
Delivery mode (*n* vaginal deliveries)	34	18
Feeding at 2, 10, 30 and 90 days (*n* exclusive breast-feding)	27, 27, 26, and 25	41, 40, 21, and 13

## Data Availability

The raw sequences reported in this article have been deposited in the NCBI Short Read Archive (SRA) under bioproject codes PRJNA616077 and PRJNA691711.

## Supplementary Materials

The following are available online at https://www.mdpi.com/1422-0067/22/7/3382/s1, Figure S1: Fecal levels of the dominant *Bifidobacterium* species and total bifidobacteria in full-term and preterm babies during the first three months of life as determined by qPCR. Figure S2: Fecal levels of the dominant *Bifidobacterium* species and total bifidobacteria in vaginally delivered and CS-delivered full-term babies during the first three months of life as determined by qPCR. Figure S3: Heat map showing the relative proportions of the different *Bifidobacterium* species/subspecies at two, 10, 30, and 90 days of age in exclusively breast-fed or formula/mixed fed vaginally delivered full-term babies. Figure S4: Fecal levels of the dominant *Bifidobacterium* species and total bifidobacteria in vaginally delivered full-term babies being exclusively breast-fed or under formula/mixed-feeding, during the first three months of life as determined by qPCR. Table S1: Occurrence of the different *Bifidobacterium* species/subspecies according to the ITS sequencing results (% of infants in which sequences from the species/subspecies were detected) at the different time points analyses (two, 10, 30, and 90 days of age) in the infant cohort included in this study. Table S2: Relative proportion of the minority *Bifidobacterium* species in the feces of full-term and preterm neonates at two, 10, 30, and 90 days of age. Table S3: Relative proportion of the *Bifidobacterium* species in the feces of vaginally delivered and Cs-delivered full-term neonates at two, 10, 30, and 90 days of age. Table S4: Relative proportion of the *Bifidobacterium* species in the feces of vaginally delivered and CS-delivered preterm neonates at two, 10, 30, and 90 days of age.
