# Supplementary material for: Early-Life Development of the Bifidobacterial Community in the Infant Gut
Source: Int J Mol Sci. 2021 Mar 25;22(7):3382. doi: 10.3390/ijms22073382 (PMC8036440; doi:10.3390/ijms22073382)
Supplement: Supplementary file 1 [file ijms-22-03382-s001.pdf]

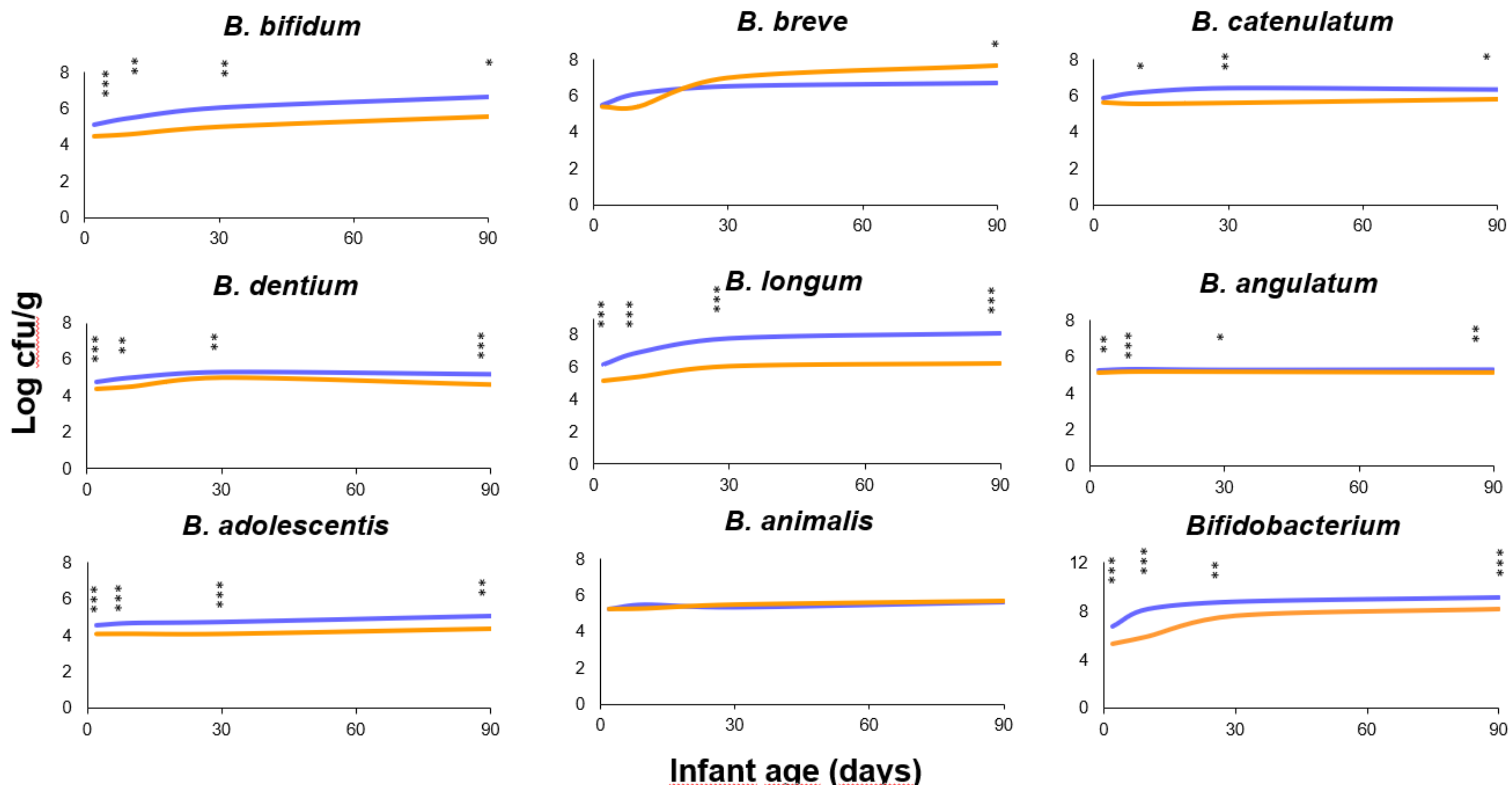

Figure S1. qPCR fecal levels (Log Nº cells/gr) of the dominant Bifidobacterium species (*B. longum, B. bifidum, B. breve, B. catenulatum, B, dentium, B. adolescentis,* B. *animalis* subsp. *lactis*, *B. angulatum* and total *Bifidobacterium*) in full-term (blue line) and preterm babies (orange line) during the first three months of life. * p<0.05, ** p<0.01, ***p<0.001.

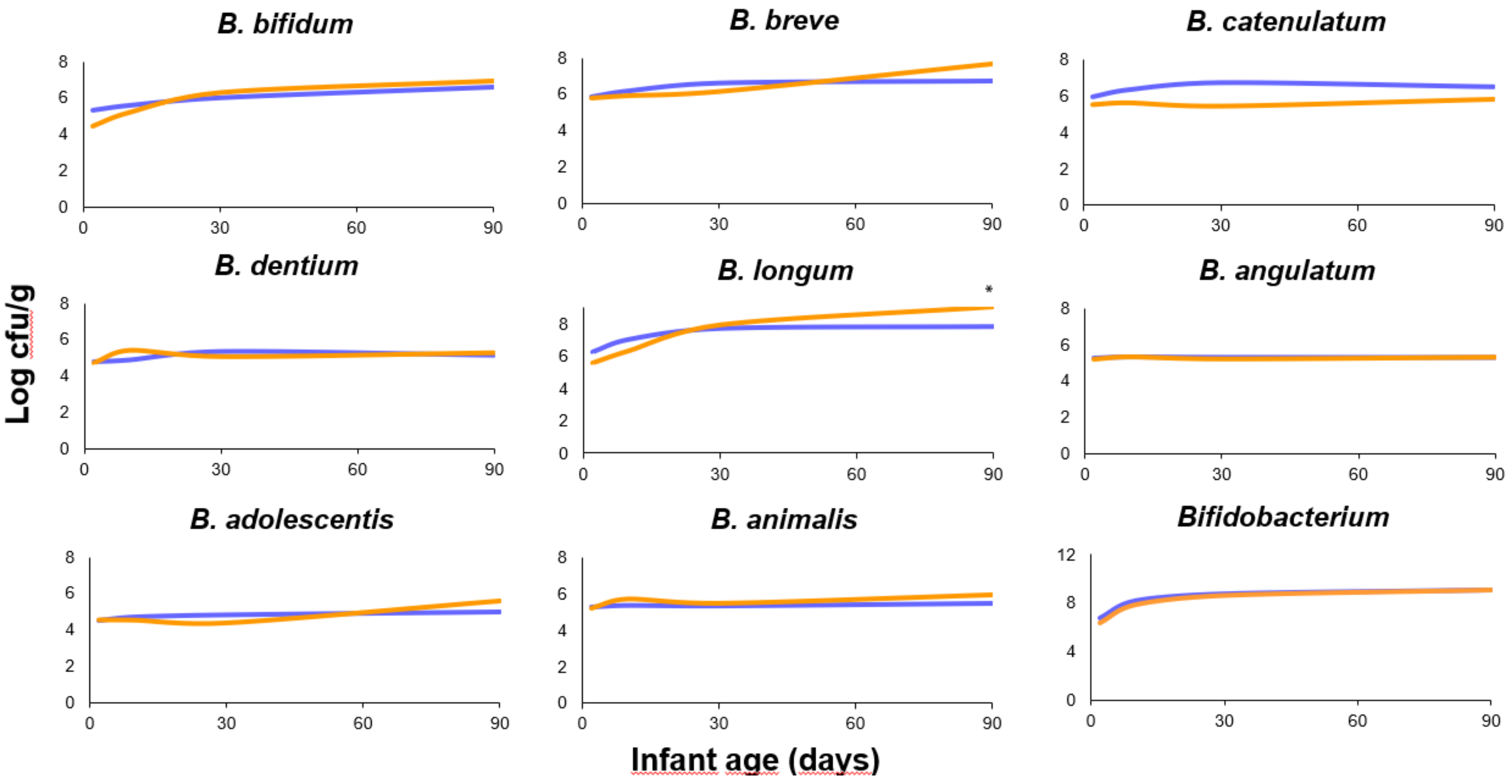

Figure S2. Fecal levels (Log Nº cells/gr) of *B. longum*, *B. bifidum*, *B. breve*, *B. catenulatum*, *B, dentium*, *B. adolescentis*, *B. animalis* subsp. *lactis*, *B. angulatum* and total *Bifidobacterium* in vaginally delivered (blue line) and CS-delivered full-term babies (orange line) during the first three months of live. * $p<0.05$.

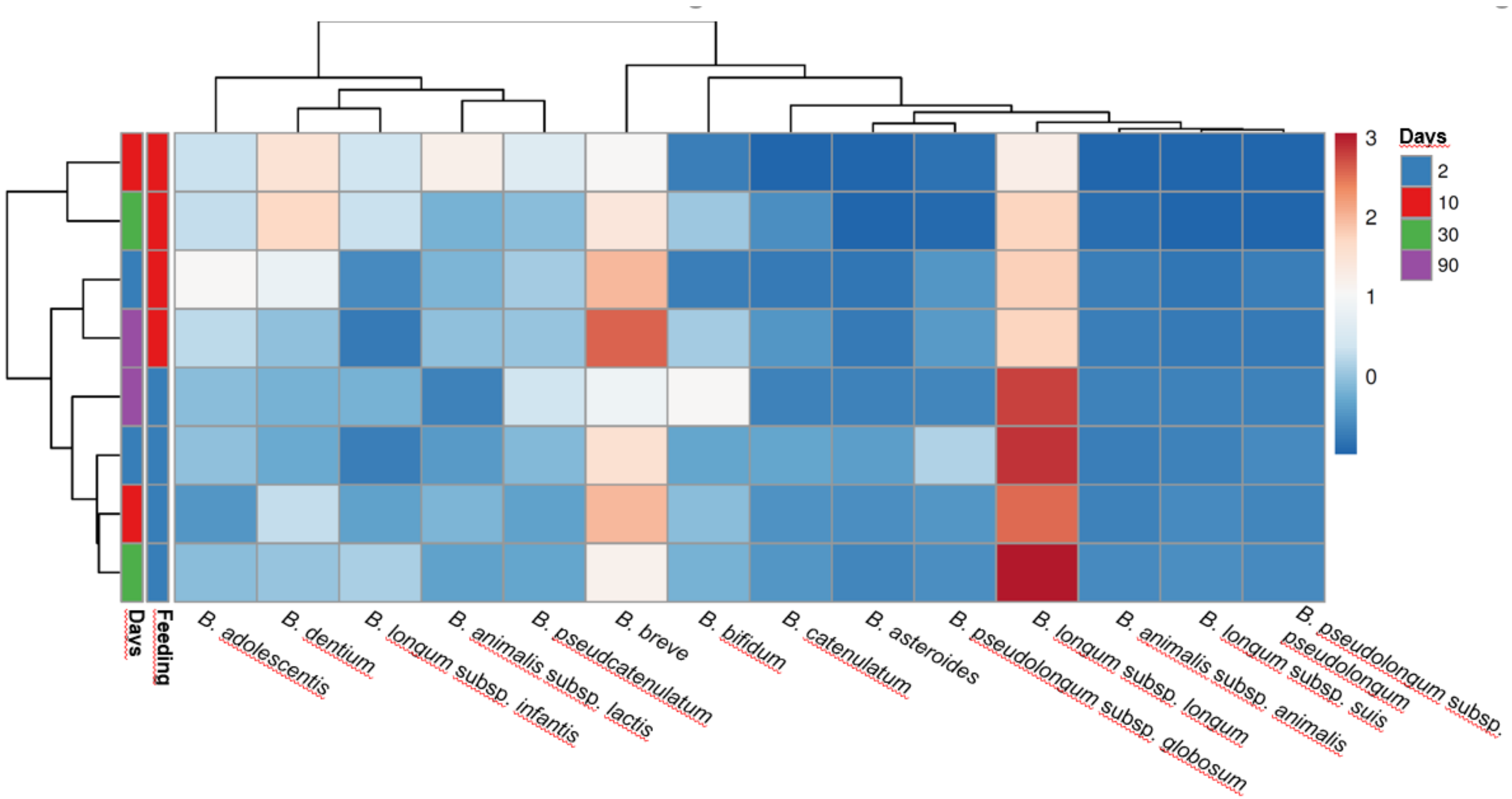

Figure S3. Heat map showing the relative proportions of the different *Bifidobacterium* species/subspecies at 2, 10, 30 and 90 days of age in vaginally delivered full-term babies exclusively breast-fed (Feeding: blue) or received formula/mixed feeding (Feeding: red).

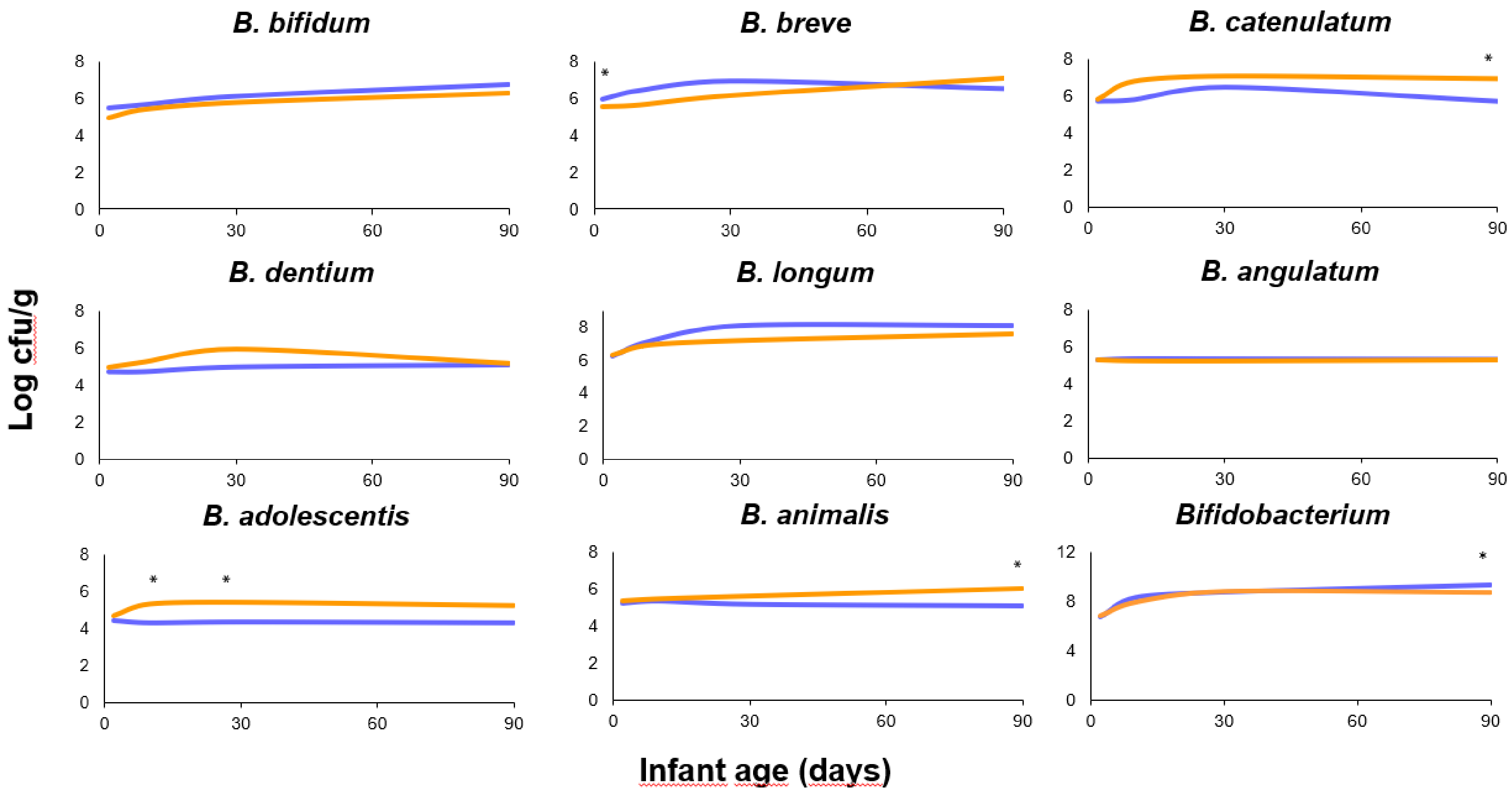

Figure S4. Fecal levels (Log Nº cells/gr) of *B. longum, B. bifidum, B. breve, B. catenulatum, B, dentium, B. adolescentis, B. animalis subsp*. lactis, *B. angulatum* and total *Bifidobacterium* in vaginally delivered full-term babies being exclusively breastfed (blue line) or under formula/mix-feeding (orange line), during the first three months of life. * $p<0.05$

Table S1. Occurrence of the different *Bifidobacterium* species/subspecies according to the ITS sequencing results (% of infants in which sequences from the specie/sub-specie were detected) at the different time points analyses (2, 10, 30 and 90 days of age) in the infant cohort included in this study.

	**2 days**	**10 days**	**30 days**	**90 days**
B. adolescentis	84.3	85.5	84.6	85.2
B. animalis* subsp. *animalis	38.6	48.2	35.9	39.3
B. animalis* subsp. *lactis	79.5	74.7	83.3	88.5
B. anseris	30.1	31.1	26.9	23.0
B. asteroides	42.2	41.0	44.9	44.3
B. biavatii	15.7	18.1	12.8	14.8
B. bifidum	78.3	79.5	82.1	83.6
B. breve	96.4	97.6	96.6	98.4
B. catenulatum	63.9	61.4	50	52.5
B. choerinum	25.3	25.3	21.5	19.7
B. crudilactis	31.3	34.9	33.3	31.1
B. dentium	73.5	73.5	71.8	78.7
B. eulemuris	20.5	14.5	7.7	6.6
B. italicum	22.9	30.1	21.8	23.0
B. kashiwanohense	42.2	39.8	37.2	41.0
B. longum* subsp. *infantis	65.1	63.9	62.8	55.7
B. longum* subsp. *longum	97.6	98.8	98.7	98.4
B. longum* subsp. *suis	62.7	61.4	62.8	70.5
B. longum subspp	80.7	68.7	62.8	70.5
B. magnum	41.0	41.0	37.2	44.3
B. mongoliense	36.1	37.3	35.9	36.1
B. parmae	36.1	33.7	29.5	21.3
B. pseudocatenulatum	73.5	69.9	67.9	70.5
B. pseudolongum* subsp. *globosum	66.3	71.1	73.1	72.1
B. pseudolongum* subsp. *pseudolongum	57.8	50.6	60.3	55.7
***B. pseudolongum* subsp**	54.2	48.2	42.3	41.0
B. reuteri	28.9	24.1	29.5	37.7
B. scardovii	22.9	20.5	26.9	29.5
B. stellenboschense	22.0	14.5	15.4	11.5
B. thermacidophilum* subsp. *porcinum	24.1	31.3	20.5	19.7

B. thermacidophilum* subsp. *thermacidophilum	25.3	24.1	23.1	23.0
B. thermophilum	36.1	38.6	33.3	36.1
B. tissieri	8.4	13.3	15.4	8.2
B. tsurumiense	19.3	11.8	15.4	14.8
B. vansinderenii	22.9	22.9	20.5	23.0
**Others**	63.9	59	61.5	59.0
**Putative new bifidobacterial species**	89.2	90.9	87.2	93.4

Table S2. Relative proportion (%) of the minority *Bifidobacterium* species in the faeces of full-term and preterm neonates at 2, 10, 30 and 90 days of age. ns: non statistically significant differences (p>0.05).

		**2 d**		**10 d**		**30 d**		**90 d**	
		Mean ± SD	*p*-value	Mean ± SD	*p*-value	Mean ± SD	*p*-value	Mean ± SD	*p*-value
B. animalis* subsp. *animalis	TERM	0.265 ± 0.701	ns	0.028 ± 0.112	0.008	0.286 ± 1.569	ns	0.256 ± 1.203	ns
	PRETERM	0.060 ± 0.191		0.127 ± 0.370		0.053 ± 0.253		0.006 ± 0.013	
B. anseris	TERM	0.010 ± 0.067	0.000	0.006 ± 0.255	0.000	0.006 ± 0.255	0.000	0.001 ± 0.006	0.000
	PRETERM	0.350 ± 1.143		0.367 ± 0.718		0.015 ± 0.090		0.029 ± 0.051	
B. biavatii	TERM	0.001 ± 0.003	ns	0.003 ± 0.011	0.006	0.003 ± 0.011	ns	0.002 ± 0.009	ns
	PRETERM	0.025 ± 0.067		0.053 ± 0.152		0.082 ± 0.269		0.003 ± 0.009	
B. choerinum	TERM	0.004 ± 0.024	0.000	0.003 ± 0.020	0.000	0.001 ± 0.003	0.035	0.001 ± 0.002	0.007
	PRETERM	0.088 ± 0.230		0.101 ± 0.203		0.063 ± 0.194		0.012 ± 0.029	
B. crudilactis	TERM	0.147 ± 0.914	0.000	0.021 ± 0.093	0.000	0.000 ± 0.001	0.000	0.004 ± 0.017	0.000
	PRETERM	0.169 ± 0.230		0.294 ± 0.685		0.116 ± 0.177		0.071 ± 0.148	
B. eulemuris	TERM	0.056 ± 0.208	ns	0.013 ± 0.078	ns	0.034 ± 0.197	ns	0.000 ± 0.001	ns
	PRETERM	0.264 ± 0.978		0.219 ± 1.032		0.731 ± 4.506		0.001 ± 0.004	
B. italicum	TERM	0.002 ± 0.012	0.000	0.002 ± 0.009	0.000	0.000 ± 0.001	0.000	0.000 ± 0.001	0.000
	PRETERM	0.053 ± 0.094		0.912 ± 5.528		0.026 ± 0.052		0.366 ± 1.765	
B. kashiwanohense	TERM	0.502 ± 2.046	ns	0.059 ± 0.195	0.025	0.747 ± 4.335	ns	0.515 ± 2.726	ns
	PRETERM	0.048 ± 0.097		0.036 ± 0.085		0.018 ± 0.043		0.038 ± 0.108	
B. longum* subsp. *suis	TERM	0.296 ± 1.447	0.000	0.480 ± 2.186	0.000	0.569 ± 2.944	0.001	0.105 ± 0.311	0.000
	PRETERM	1.671 ± 1.987		2.110 ± 3.372		1.517 ± 5.523		1.627 ± 5.897	
***B. longum* subspp**	TERM	0.023 ± 0.032	0.028	0.037 ± 0.150	0.000	0.047 ± 0.201	ns	0.018 ± 0.034	0.047
	PRETERM	0.044 ± 0.061		0.037 ± 0.043		0.072 ± 0.280		0.109 ± 0.390	
B. magnum	TERM	0.002 ± 0.006	0.000	0.045 ± 0.262	0.000	0.002 ± 0.005	0.000	0.009 ± 0.032	0.002

	PRETERM	0.400 ± 0.534		0.305 ± 0.474		0.280 ± 0.555		0.117 ± 0.308	
B. mongoliense	TERM	0.015 ± 0.068	0.000	0.004 ± 0.019	0.000	0.054 ± 0.291	0.003	0.012 ± 0.063	0.001
	PRETERM	0.116 ± 0.208		0.118 ± 0.259		0.069 ± 0.112		0.042 ± 0.084	
B. parmae	TERM	1.197 ± 4.616	ns	0.058 ± 0.195	0.044	0.019 ± 0.066	0.032	0.001 ± 0.006	0.013
	PRETERM	0.434 ± 2.327		0.687 ± 3.847		0.489 ± 1.926		0.144 ± 0.359	
***B. pseudolongum* subsp**	TERM	0.613 ± 2.623	0.000	0.119 ± 0.653	0.000	0.002 ± 0.007	0.000	0.026 ± 0.117	0.001
	PRETERM	0.697 ± 1.318		1.333 ± 2.342		0.676 ± 1.490		0.463 ± 1.303	
B. reuteri	TERM	0.035 ± 0.143	0.038	0.047 ± 0.181	0.004	0.016 ± 0.080	ns	0.018 ± 0.071	0.004
	PRETERM	0.169 ± 0.332		0.070 ± 0.175		0.043 ± 0.127		0.036 ± 0.055	
B. scardovii	TERM	0.041 ± 0.180	ns	0.015 ± 0.061	ns	0.737 ± 4.633	ns	0.379 ± 2.223	ns
	PRETERM	0.041 ± 0.157		0.023 ± 0.096		0.024 ± 0.081		0.176 ± 0.857	
B. stellenboschense	TERM	0.032 ± 0.124	ns	0.007 ± 0.045	0.048	0.000 ± 0.000	ns	0.000 ± 0.001	ns
	PRETERM	0.012 ± 0.041		0.581 ± 3.668		0.007 ± 0.023		0.002 ± 0.004	
B. thermacidophilum* subsp. *porcinum	TERM	0.003 ± 0.020	0.000	0.001 ± 0.006	0.000	0.019 ± 0.117	0.000	0.001 ± 0.004	0.000
	PRETERM	0.114 ± 0.420		1.693 ± 9.863		0.120 ± 0.442		0.019 ± 0.045	
B. thermacidophilum* subsp. *thermacidophilum	TERM	0.001 ± 0.002	0.000	0.010 ± 0.057	0.000	0.000 ± 0.001	0.000	0.001 ± 0.005	0.008
	PRETERM	0.225 ± 0.548		0.114 ± 0.217		0.117 ± 0.361		0.038 ± 0.147	
B. thermophilum	TERM	0.028 ± 0.126	0.000	0.012 ± 0.035	0.001	0.003 ± 0.009	0.005	0.007 ± 0.024	ns
	PRETERM	0.084 ± 0.146		0.071 ± 0.108		0.070 ± 0.168		0.042 ± 0.129	
B. tissieri	TERM	0.147 ± 0.940	0.045	0.000 ± 0.000	0.000	0.000 ± 0.000	0.001	0.000 ± 0.000	ns
	PRETERM	0.010 ± 0.030		0.011 ± 0.026		0.013 ± 0.034		0.038 ± 0.186	
B. tsurumiense	TERM	0.000 ± 0.001	0.002	0.001 ± 0.003	ns	0.000 ± 0.000	0.001	0.001 ± 0.002	ns
	PRETERM	0.013 ± 0.029		0.006 ± 0.021		0.884 ± 5.397		0.003 ± 0.006	
B. vansinderenii	TERM	0.022 ± 0.123	0.000	0.020 ± 0.100	0.009	0.000 ± 0.001	0.000	0.002 ± 0.008	0.000
	PRETERM	0.095 ± 0.148		0.110 ± 0.179		0.081 ± 0.137		0.105 ± 0.372	
**Putative new bifidobacterial species**	TERM	2.058 ± 7.364	0.012	1.385 ± 4.180	0.001	4.393 ± 13.733	ns	3.434 ± 9.304	ns
	PRETERM	2.239 ± 2.177		2.875 ± 3.693		1.658 ± 2.168		1.137 ± 2.700	
**Others**	TERM	0.188 ± 0.413	0.001	0.240 ± 1.056	0.000	0.086 ± 0.330	0.004	0.074 ± 0.263	0.001
	PRETERM	0.839 ± 1.260		0.632 ± 1.027		0.483 ± 0.813		0.376 ± 0.618	

Table S3. Relative proportion (%) of the *Bifidobacterium* species in the faeces of vaginally delivered and Cs-delivered full-term neonates at 2, 10, 30 and 90 days of age. ns: non statistically significant differences (p>0.05).

		2 d		10 d		30 d		90 d	
		Mean ± SD	*p*-value	Mean ± SD	*p*-value	Mean ± SD	*p*-value	Mean ± SD	*p*-value
B. animalis* subsp. *animalis	VAGINAL	0.267 ± 0.693	ns	0.036 ± 0.126	ns	0.367 ± 1.781	ns	0.319 ± 1.342	ns
	C-SECTION	0.259 ± 0.772		0.001 ± 0.002		0.008 ± 0.017		0.003 ± 0.004	
B. anseris	VAGINAL	0.013 ± 0.075	ns	0.007 ± 0.029	ns	0.000 ± 0.000	ns	0.001 ± 0.002	ns
	C-SECTION	0.000 ± 0.000		0.000 ± 0.000		0.069 ± 0.189		0.005 ± 0.014	
B. biavatii	VAGINAL	0.001 ± 0.004	ns	0.003 ± 0.012	ns	0.003 ± 0.013	ns	0.002 ± 0.010	ns
	C-SECTION	0.000 ± 0.000		0.000 ± 0.000		0.000 ± 0.000		0.000 ± 0.000	
B. choerinum	VAGINAL	0.005 ± 0.027	ns	0.004 ± 0.023	ns	0.001 ± 0.004	ns	0.001 ± 0.003	ns
	C-SECTION	0.001 ± 0.002		0.001 ± 0.001		0.001 ± 0.001		0.000 ± 0.000	
B. crudilactis	VAGINAL	0.185 ± 1.028	ns	0.010 ± 0.039	ns	0.000 ± 0.001	ns	0.005 ± 0.018	ns
	C-SECTION	0.002 ± 0.006		0.063 ± 0.189		0.001 ± 0.003		0.004 ± 0.009	
B. eulemuris	VAGINAL	0.051 ± 0.208	ns	0.017 ± 0.088	ns	0.000 ± 0.001	ns	0.000 ± 0.001	ns
	C-SECTION	0.074 ± 0.222		0.001 ± 0.002		0.149 ± 0.411		0.000 ± 0.001	
B. italicum	VAGINAL	0.002 ± 0.013	ns	0.003 ± 0.010	ns	0.000 ± 0.001	ns	0.000 ± 0.001	ns
	C-SECTION	0.000 ± 0.000		0.000 ± 0.000		0.000 ± 0.000		0.000 ± 0.000	
B. kashiwanohense	VAGINAL	0.635 ± 2.289	ns	0.054 ± 0.186	ns	0.946 ± 4.924	ns	0.642 ± 3.045	ns
	C-SECTION	0.003 ± 0.006		0.079 ± 0.236		0.060 ± 0.160		0.008 ± 0.014	
B. longum* subsp. *suis	VAGINAL	0.369 ± 1.624	ns	0.473 ± 2.427	ns	0.678 ± 3.340	ns	0.129 ± 0.344	ns
	C-SECTION	0.022 ± 0.034		0.504 ± 0.964		0.191 ± 0.448		0.010 ± 0.025	
***B. longum* subspp**	VAGINAL	0.019 ± 0.028	ns	0.039 ± 0.168	ns	0.056 ± 0.228	ns	0.019 ± 0.038	ns
	C-SECTION	0.039 ± 0.041		0.027 ± 0.048		0.016 ± 0.025		0.013 ± 0.014	
B. magnum	VAGINAL	0.002 ± 0.005	ns	0.005 ± 0.026	ns	0.001 ± 0.002	ns	0.008 ± 0.034	ns
	C-SECTION	0.004 ± 0.009		0.189 ± 0.566		0.004 ± 0.010		0.010 ± 0.025	
B. mongoliense	VAGINAL	0.019 ± 0.077	ns	0.005 ± 0.021	ns	0.068 ± 0.330	ns	0.014 ± 0.070	ns
	C-SECTION	0.000 ± 0.000		0.000 ± 0.000		0.003 ± 0.008		0.005 ± 0.011	
B. parmae	VAGINAL	1.219 ± 5.091	ns	0.073 ± 0.218	ns	0.020 ± 0.072	ns	0.002 ± 0.007	ns
	C-SECTION	1.115 ± 2.224		0.000 ± 0.000		0.014 ± 0.040		0.000 ± 0.001	
***B. pseudolongum* subspp**	VAGINAL	0.771 ± 2.938	ns	0.139 ± 0.737	ns	0.001 ± 0.003	ns	0.025 ± 0.125	ns
	C-SECTION	0.018 ± 0.053		0.045 ± 0.096		0.006 ± 0.013		0.031 ± 0.080	
B. reuteri	VAGINAL	0.043 ± 0.159	ns	0.028 ± 0.108	ns	0.019 ± 0.090	ns	0.020 ± 0.078	ns

	C-SECTION	0.003 ± 0.007		0.124 ± 0.350		0.008 ± 0.022		0.008 ± 0.020	
B. scardovii	VAGINAL	0.032 ± 0.171	ns	0.010 ± 0.049	ns	0.005 ± 0.019	ns	0.004 ± 0.010	ns
	C-SECTION	0.075 ± 0.220		0.031 ± 0.094		3.259 ± 9.768		1.879 ± 4.972	
B. stellenboschense	VAGINAL	0.040 ± 0.137	ns	0.000 ± 0.000	ns	0.000 ± 0.001	ns	0.000 ± 0.001	ns
	C-SECTION	0.000 ± 0.000		0.036 ± 0.100		0.000 ± 0.000		0.000 ± 0.000	
B. thermacidophilum* subsp. *porcinum	VAGINAL	0.004 ± 0.023	ns	0.002 ± 0.006	ns	0.000 ± 0.000	ns	0.001 ± 0.005	ns
	C-SECTION	0.000 ± 0.000		0.000 ± 0.000		0.082 ± 0.246		0.000 ± 0.000	
B. thermacidophilum* subsp. *thermacidophilum	VAGINAL	0.001 ± 0.002	ns	0.013 ± 0.064	ns	0.000 ± 0.001	ns	0.002 ± 0.005	ns
	C-SECTION	0.000 ± 0.000		0.000 ± 0.000		0.000 ± 0.001		0.000 ± 0.000	
B. thermophilum	VAGINAL	0.035 ± 0.141	ns	0.006 ± 0.019	ns	0.003 ± 0.010	ns	0.008 ± 0.027	ns
	C-SECTION	0.001 ± 0.003		0.034 ± 0.065		0.002 ± 0.004		0.002 ± 0.003	
B. tissieri	VAGINAL	0.182 ± 1.048	ns	0.000 ± 0.000	ns	0.000 ± 0.000	ns	0.000 ± 0.000	ns
	C-SECTION	0.000 ± 0.000		0.000 ± 0.000		0.000 ± 0.000		0.000 ± 0.000	
B. tsurumiense	VAGINAL	0.000 ± 0.001	ns	0.001 ± 0.003	ns	0.000 ± 0.000	ns	0.001 ± 0.003	ns
	C-SECTION	0.001 ± 0.003		0.000 ± 0.000		0.000 ± 0.000		0.000 ± 0.000	
B. vansinderenii	VAGINAL	0.024 ± 0.137	ns	0.020 ± 0.110	ns	0.000 ± 0.001	ns	0.000 ± 0.002	ns
	C-SECTION	0.014 ± 0.040		0.018 ± 0.050		0.000 ± 0.000		0.007 ± 0.018	
**Putative new bifidobacterial species**	VAGINAL	2.415 ± 8.244	ns	1.515 ± 4.363	ns	5.544 ± 15.456	ns	4.230 ± 10.281	ns
	C-SECTION	0.710 ± 1.326		0.909 ± 1.800		0.430 ± 0.905		0.250 ± 0.357	
**Others**	VAGINAL	0.174 ± 0.371	ns	0.104 ± 0.335	ns	0.108 ± 0.373	ns	0.088 ± 0.293	ns
	C-SECTION	0.240 ± 0.568		0.740 ± 2.215		0.012 ± 0.025		0.015 ± 0.029	

Table S4. Relative proportion (%) of the *Bifidobacterium* species in the faeces of vaginally delivered and CS-delivered preterm neonates at 2, 10, 30 and 90 days of age. ns: non statistically significant differences (p>0.05).

		**2 d**		**10 d**		**30 d**		**90 d**	
		Mean ± SD	*p*-value	Mean ± SD	*p*-value	Mean ± SD	*p*-value	Mean ± SD	*p*-value
B. longum* subsp. *longum	**VAGINAL**	25.330 ± 27.040	ns	24.160 ± 28.644	ns	15.880 ± 22.914	ns	11.200 ± 14.490	ns
	C-SECTION	26.820 ± 29.080		23.600 ± 28.724		11.900 ± 15.452		9.730 ± 13.380	
B. breve	VAGINAL	19.930 ± 9.808	ns	11.590 ± 10.033	ns	36.490 ± 31.858	ns	41.500 ± 28.200	ns
	C-SECTION	18.390 ± 21.720		21.420 ± 26.048		37.130 ± 37.192		53.600 ± 31.850	
B. pseudolongum* subsp. *globosum	VAGINAL	10.190 ± 9.562	ns	16.490 ± 16.720	ns	13.500 ± 15.340	0.036	6.230 ± 9.879	ns
	C-SECTION	10.960 ± 12.150		10.480 ± 13.250		5.143 ± 7.870		2.120 ± 3.434	
B. animalis* subsp. *lactis	VAGINAL	8.367 ± 5.717	ns	6.273 ± 6.165	ns	2.918 ± 3.544	ns	3.790 ± 5.644	ns
	C-SECTION	10.500 ± 9.995		9.493 ± 16.703		3.691 ± 4.722		3.920 ± 8.229	
B. asteroides	VAGINAL	5.363 ± 6.792	0.019	5.448 ± 11.418	ns	2.784 ± 3.856	0.029	2.760 ± 4.442	ns
	C-SECTION	1.761 ± 3.343		3.574 ± 7.113		1.735 ± 5.373		1.830 ± 3.954	
B. bifidum	VAGINAL	4.535 ± 4.882	0.005	2.791 ± 4.613	ns	2.425 ± 4.166	ns	1.160 ± 1.045	ns
	C-SECTION	1.614 ± 2.905		2.730 ± 3.900		3.935 ± 7.293		2.780 ± 5.434	
B. adolescentis	VAGINAL	4.418 ± 3.897	ns	4.663 ± 5.907	ns	2.309 ± 4.030	ns	0.449 ± 0.500	ns
	C-SECTION	10.650 ± 17.440		8.523 ± 13.085		7.069 ± 13.314		5.450 ± 14.600	
B.pseudolongum* subsp. *pseudolongum	VAGINAL	4.122 ± 4.523	ns	5.327 ± 5.404	ns	3.885 ± 6.055	ns	0.972 ± 1.171	ns
	C-SECTION	5.083 ± 7.500		3.158 ± 4.272		1.841 ± 4.315		0.756 ± 1.266	
B. dentium	VAGINAL	3.902 ± 11.140	ns	6.130 ± 21.689	ns	5.296 ± 11.309	ns	15.200 ± 31.590	ns
	C-SECTION	4.047 ± 10.890		2.402 ± 6.040		10.510 ± 26.145		4.600 ± 17.050	
B. pseudocatenulatum	VAGINAL	3.102 ± 9.282	ns	3.503 ± 12.752	ns	7.007 ± 23.518	ns	10.200 ± 20.290	ns
	C-SECTION	2.059 ± 5.273		0.384 ± 0.574		0.603 ± 1.077		5.180 ± 13.180	
B. longum* subsp. *suis	VAGINAL	1.404 ± 1.085	ns	1.677 ± 3.580	ns	0.421 ± 0.469	ns	0.705 ± 1.492	ns
	C-SECTION	1.868 ± 2.459		2.417 ± 3.258		2.314 ± 7.211		2.200 ± 7.463	
***B .pseudolongum* subspp**	VAGINAL	0.904 ± 1.728	ns	1.428 ± 2.250	ns	0.981 ± 1.729	ns	0.756 ± 1.902	ns
	C-SECTION	0.543 ± 0.922		1.266 ± 2.450		0.455 ± 1.286		0.280 ± 0.751	
B. longum* subsp. *infantis	VAGINAL	0.668 ± 1.288	ns	0.508 ± 1.151	ns	0.222 ± 0.693	ns	0.059 ± 0.139	ns
	C-SECTION	0.576 ± 1.102		0.340 ± 0.617		7.653 ± 23.550		5.820 ± 16.170	
B. magnum	VAGINAL	0.654 ± 0.600	0.005	0.266 ± 0.372	ns	0.297 ± 0.545	ns	0.073 ± 0.070	ns
	C-SECTION	0.212 ± 0.397		0.333 ± 0.541		0.267 ± 0.575		0.144 ± 0.391	
B. catenulatum	VAGINAL	0.428 ± 0.452	ns	1.092 ± 2.349	ns	0.120 ± 0.148	ns	0.118 ± 0.307	ns

	C-SECTION	0.299 ± 0.444		0.404 ± 0.755		0.106 ± 0.213		0.101 ± 0.230	
B. thermacidophilum* subsp. *thermacidophilum	VAGINAL	0.408 ± 0.764	ns	0.112 ± 0.246	ns	0.149 ± 0.526	ns	0.018 ± 0.023	ns
	C-SECTION	0.090 ± 0.251		0.114 ± 0.200		0.093 ± 0.174		0.050 ± 0.188	
B. reuteri	VAGINAL	0.284 ± 0.433	ns	0.103 ± 0.226	0.050	0.094 ± 0.186	0.024	0.045 ± 0.050	ns
	C-SECTION	0.085 ± 0.204		0.046 ± 0.127		0.006 ± 0.021		0.030 ± 0.055	
B. crudilactis	VAGINAL	0.228 ± 0.273	ns	0.191 ± 0.234	ns	0.182 ± 0.216	0.031	0.105 ± 0.198	ns
	C-SECTION	0.125 ± 0.187		0.368 ± 0.875		0.069 ± 0.127		0.050 ± 0.108	
B. mongoliense	VAGINAL	0.215 ± 0.283	0.006	0.191 ± 0.382	ns	0.068 ± 0.098	ns	0.042 ± 0.055	ns
	C-SECTION	0.043 ± 0.074		0.067 ± 0.091		0.070 ± 0.123		0.042 ± 0.100	
B. thermophilum	VAGINAL	0.127 ± 0.159	ns	0.048 ± 0.070	ns	0.077 ± 0.171	ns	0.081 ± 0.195	ns
	C-SECTION	0.051 ± 0.130		0.086 ± 0.127		0.064 ± 0.169		0.017 ± 0.057	
B. anseris	VAGINAL	0.122 ± 0.136	ns	0.516 ± 0.928	ns	0.067 ± 0.073	ns	0.066 ± 0.067	0.012
	C-SECTION	0.519 ± 1.494		0.262 ± 0.521		0.566 ± 1.728		0.006 ± 0.010	
B. vansinderenii	VAGINAL	0.114 ± 0.151	ns	0.139 ± 0.173	ns	0.083 ± 0.971	ns	0.243 ± 0.589	ns
	C-SECTION	0.081 ± 0.147		0.089 ± 0.184		0.079 ± 0.162		0.019 ± 0.035	
B. scardovii	VAGINAL	0.095 ± 0.234	ns	0.046 ± 0.147	ns	0.025 ± 0.085	ns	0.444 ± 1.382	ns
	C-SECTION	0.002 ± 0.005		0.008 ± 0.020		0.022 ± 0.080		0.008 ± 0.026	
B. kashiwanohense	VAGINAL	0.089 ± 0.131	ns	0.030 ± 0.054	ns	0.033 ± 0.061	ns	0.044 ± 0.104	ns
	C-SECTION	0.018 ± 0.045		0.040 ± 0.103		0.008 ± 0.017		0.034 ± 0.113	
B. eulemuris	VAGINAL	0.064 ± 0.201	ns	0.386 ± 1.558	ns	1.736 ± 6.944	ns	0.002 ± 0.007	ns
	C-SECTION	0.412 ± 1.270		0.101 ± 0.358		0.000 ± 0.000		0.000 ± 0.001	
B. parmae	VAGINAL	0.059 ± 0.103	ns	0.096 ± 0.290	ns	0.053 ± 0.194	ns	0.010 ± 0.022	ns
	C-SECTION	0.711 ± 3.066		1.105 ± 5.024		0.806 ± 2.501		0.227 ± 0.442	
B. italicum	VAGINAL	0.044 ± 0.108	ns	0.057 ± 0.080	ns	0.015 ± 0.024	ns	0.933 ± 2.842	ns
	C-SECTION	0.060 ± 0.084		1.517 ± 7.227		0.034 ± 0.065		0.011 ± 0.029	
B. choerinum	VAGINAL	0.042 ± 0.128	ns	0.098 ± 0.183	ns	0.029 ± 0.068	ns	0.010 ± 0.022	ns
	C-SECTION	0.122 ± 0.282		0.103 ± 0.220		0.088 ± 0.248		0.013 ± 0.033	
B. animalis* subsp. *animalis	VAGINAL	0.041 ± 0.066	ns	0.090 ± 0.230	ns	0.010 ± 0.020	ns	0.007 ± 0.011	ns
	C-SECTION	0.074 ± 0.247		0.154 ± 0.446		0.084 ± 0.332		0.005 ± 0.015	
B. biavatii	VAGINAL	0.041 ± 0.093	ns	0.058 ± 0.184	ns	0.036 ± 0.136	ns	0.006 ± 0.014	ns
	C-SECTION	0.013 ± 0.036		0.050 ± 0.128		0.115 ± 0.333		0.001 ± 0.004	
***B. longum* subspp**	VAGINAL	0.035 ± 0.033	ns	0.037 ± 0.041	ns	0.021 ± 0.031	ns	0.024 ± 0.027	ns

	C-SECTION	0.050 ± 0.076		0.038 ± 0.045		0.109 ± 0.366		0.161 ± 0.495	
B. stellenboschense	VAGINAL	0.018 ± 0.056	ns	1.390 ± 5.696	ns	0.011 ± 0.033	ns	0.003 ± 0.005	ns
	C-SECTION	0.007 ± 0.026		0.009 ± 0.029		0.005 ± 0.012		0.001 ± 0.003	
B. thermacidophilum* subsp. *porcinum	VAGINAL	0.029 ± 0.055	ns	0.218 ± 0.445	ns	0.032 ± 0.049	ns	0.012 ± 0.016	ns
	C-SECTION	0.177 ± 0.548		2.738 ± 12.896		0.184 ± 0.577		0.023 ± 0.057	
B. tsurumiense	VAGINAL	0.027 ± 0.040	0.034	0.004 ± 0.010	ns	0.014 ± 0.035	ns	0.005 ± 0.007	ns
	C-SECTION	0.003 ± 0.007		0.008 ± 0.027		1.516 ± 7.094		0.001 ± 0.005	
B. tissieri	VAGINAL	0.009 ± 0.036	ns	0.010 ± 0.025	ns	0.002 ± 0.005	ns	0.001 ± 0.004	ns
	C-SECTION	0.011 ± 0.025		0.012 ± 0.027		0.021 ± 0.043		0.061 ± 0.238	
**Putative new bifido-bacterial species**	VAGINAL	3.646 ± 2.115	0.001	4.104 ± 4.871	ns	2.088 ± 2.639	ns	2.120 ± 4.125	ns
	C-SECTION	1.335 ± 1.772		2.005 ± 2.302		1.346 ± 1.751		0.523 ± 0.949	
**Others**	VAGINAL	1.121 ± 1.146	ns	0.732 ± 0.991	ns	0.649 ± 0.988	ns	0.564 ± 0.659	ns
	C-SECTION	0.631 ± 1.325		0.561 ± 1.068		0.363 ± 0.657		0.259 ± 0.581	
